# A rare case of acute respiratory distress syndrome due to chlorine gas inhalation: Rapid progression with favorable ICU outcome

**DOI:** 10.1016/j.rmcr.2024.102148

**Published:** 2024-12-06

**Authors:** Dung Thai Pham, Bang Ngoc Dao, Dung Tien Nguyen, Ba Van Dang, Dung Tien Le, Hung Manh Do, Loc Tich Hoang, Minh Tuan Ngo, Duong Minh Vu

**Affiliations:** a Intensive Care, Emergency and Poison Control Center, Military Hospital 103, Vietnam Military Medical University, Viet Nam; b Respiratory Center, Military Hospital 103, Vietnam Military Medical University, Viet Nam; c Radiology Center, Military Hospital 103, Vietnam Medical Military University, Viet Nam

**Keywords:** Chlorine, Inhalation, Exposure, ARDS

## Abstract

Acute respiratory distress syndrome (ARDS) secondary to chlorine gas inhalation is a rare yet critical condition that can lead to severe respiratory failure if not managed promptly. This case report presents a 43-year-old male who developed ARDS after accidental exposure to chlorine gas during pool maintenance. The patient's condition deteriorated rapidly, with symptoms progressing to acute pulmonary edema and severe hypoxemia, requiring immediate transfer to the intensive care unit (ICU). Initial treatment included non-invasive ventilation, but the patient soon required tracheal intubation and mechanical ventilation. Despite the rapid disease progression, the patient responded well to aggressive ICU management, including oxygen therapy, mechanical ventilation, and pharmacological support. Remarkably, within seven days, the patient fully recovered and was discharged in stable condition. This case highlights the potential for a good prognosis in ARDS due to chlorine gas inhalation compared to other etiologies, emphasizing the importance of timely intervention and specialized care in the ICU.

## Highlights

• Chlorine gas inhalation rarely needs to treat in ICU.
• ARDS due to chlorine gas inhalation has better prognosis than other causes like trauma, bacterial.
• Chlorine inhalation poisoning can be fatal, but if treated promptly, even if it progresses to ARDS, a patient can still be rescued.

## 1 Introduction

Chlorine gas exposure, a significant occupational hazard in industries involving water treatment and cleaning services, can lead to severe respiratory complications including ARDS [1,2]. The pathophysiology involves a rapid dissolution of chlorine gas in the airway surface fluid, forming acids that trigger direct chemical injury to the respiratory epithelium[3,4]. This initial damage initiates an inflammatory cascade characterized by neutrophil infiltration and cytokine release, ultimately disrupting the alveolar-capillary barrier and leading to protein-rich edema in the alveolar space[5,6]. While chlorine-induced ARDS can be severe, the potential reversibility of the initial chemical injury, combined with appropriate intensive care management, may offer better outcomes compared to ARDS from other etiologies[7,8]. This case report details the successful management of severe chlorine-induced ARDS, highlighting the importance of understanding both the underlying pathophysiology and the principles of therapeutic intervention in such cases.

## 3 Discussion

Acute respiratory distress syndrome (ARDS) secondary to chlorine gas inhalation is a rare but serious complication, often requiring intensive care. While chlorine gas exposure typically results in mild to moderate respiratory symptoms that can be managed in non-ICU settings [9,10], severe cases can progress to life-threatening ARDS, necessitating advanced respiratory support. This case emphasizes several important clinical insights related to the management and prognosis of ARDS caused by chlorine gas inhalation.

Firstly, the need for ICU treatment in chlorine gas inhalation cases is uncommon [11]. Chlorine is a highly reactive gas that causes chemical irritation primarily to the respiratory tract. Inhalation typically results in symptoms such as coughing, throat irritation, and shortness of breath, which are generally self-limiting. However, in certain individuals or with prolonged exposure, the gas can cause significant lung injury, leading to ARDS [11,12]. The decision to admit such patients to the ICU is based on the severity of the respiratory failure, which is not a typical progression in most cases. In this case, the patient required ICU care due to the development of ARDS, highlighting the unpredictability of the disease course and the necessity of vigilant monitoring in cases of significant chlorine exposure.

Secondly, ARDS due to chlorine gas inhalation generally has a more favorable prognosis compared to ARDS caused by other etiologies, such as trauma or bacterial infections [13]. Chlorine-induced ARDS is primarily the result of direct chemical injury rather than ongoing systemic inflammation or infection. As such, once the initial inflammatory response is controlled and the patient is provided with adequate respiratory support, the lungs are often capable of recovering more quickly. Studies suggest that chlorine gas-induced ARDS tends to resolve faster, with lower mortality rates, compared to ARDS from causes like sepsis or blunt trauma, which are often complicated by multi-organ failure and persistent inflammatory processes. The patient in this case demonstrated significant improvement following prompt intervention, further supporting the notion that chlorine-induced ARDS carries a relatively better prognosis when managed effectively.

Finally, while chlorine inhalation can indeed be fatal, early recognition and timely intervention can significantly improve outcomes, even if the exposure progresses to ARDS. Chlorine gas is highly corrosive to the respiratory epithelium, and in severe cases, can lead to life-threatening complications such as ARDS or pulmonary edema [14]. However, with rapid initiation of appropriate treatment—such as oxygen therapy, mechanical ventilation if necessary, and anti-inflammatory agents—patients can recover fully. The favorable outcome in our case was likely influenced by several exposure-related factors. The incident occurred in an outdoor swimming pool setting, allowing for rapid gas dispersal, and the patient's immediate recognition of chlorine's irritant effects prompted quick withdrawal from the exposure area, limiting the duration of exposure. These circumstances, combined with prompt medical intervention, likely contributed to the patient's successful recovery despite developing ARDS.

In conclusion, while ARDS resulting from chlorine gas inhalation is rare, it requires prompt recognition and intensive treatment when it occurs. The prognosis in these cases is generally favorable compared to ARDS from other causes, provided that early and appropriate medical care is administered. This case underscores the critical role of timely ICU care in rescuing patients from severe chlorine inhalation injuries, even when they progress to ARDS.

## CRediT authorship contribution statement

**Dung Thai Pham:** Writing – original draft, Visualization, Supervision, Project administration, Investigation. **Bang Ngoc Dao:** Validation, Supervision, Conceptualization. **Dung Tien Nguyen:** Methodology, Investigation, Conceptualization. **Ba Van Dang:** Validation, Investigation. **Dung Tien Le:** Investigation. **Hung Manh Do:** Investigation. **Loc Tich Hoang:** Investigation. **Minh Tuan Ngo:** Visualization, Software. **Duong Minh Vu:** Writing – review & editing, Writing – original draft, Supervision, Project administration.

## Informed consent

The patient provided informed consent for the publication of this case report.

## 2 Case presentation

A 43-year-old male, employed as an outdoor swimming pool cleaner with no known history of respiratory or chronic illnesses, was admitted to the hospital due to acute shortness of breath following chlorine gas inhalation. The incident occurred at night when the patient mixed TCCA 90 % (Trichloroisocyanuric acid) and Aquafit chlorine powder 70 % (Calcium hypochlorite) during pool maintenance. Immediately after inhalation, he experienced dizziness, shortness of breath, coughing with increased phlegm production, and a runny nose. His condition rapidly deteriorated, leading to severe shortness of breath, a productive cough with pink, frothy sputum, chest pain, and epigastric discomfort, though notably without fever. He was transported to the hospital approximately 3 h later.

On arrival, the patient was conscious and afebrile, with a body temperature of 36.8 °C. However, he displayed cyanosis around his lips and extremities and was diaphoretic. He exhibited signs of acute pulmonary edema, as evidenced by the use of accessory respiratory muscles and spontaneous breathing. Oxygen therapy at 8 L per minute via a non-rebreather mask was initiated, though his oxygen saturation remained low at 90–91 %. His vital signs included a heart rate of 112 beats per minute, blood pressure of 102/70 mmHg, and a respiratory rate of 33 breaths per minute.

**Fig. 1:**
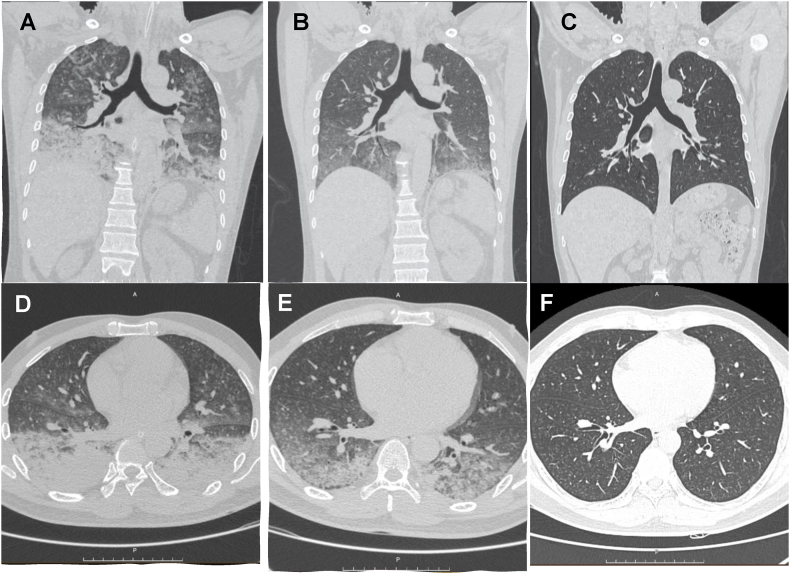
**A, B, C**: Coronal CT scans from Day 0, Day 2, and Day 5. **D, E, F**: Axial CT scans from Day 0, Day 2, and Day 5. The CT images show diffuse consolidation in both lungs, primarily in the lower regions on Day 0, indicative of acute lung injury, such as ARDS. By Day 2, the lesions exhibit rapid absorption, suggesting a favorable response to treatment. By Day 5, the lesions have nearly resolved, indicating significant recovery and resolution of lung injury.

A lung ultrasound revealed multiple B-lines throughout both lungs, strongly suggesting pulmonary edema. A chest CT scan showed consolidation and ground-glass opacities radiating from the hilum, particularly affecting the lower regions of both lungs (Fig. 1A–D). Follow-up CT scans demonstrated rapid absorption of lesions by Day 2 (Fig. 1B–E) and near-complete resolution by Day 5 (Fig. 1C–F), correlating with the patient's clinical improvement. An ECG revealed sinus tachycardia at 119 beats per minute, with no other abnormalities. An echocardiogram confirmed a structurally normal heart with good function.

Initial blood work demonstrated hemoglobin levels of 138 g/L, a white blood cell count of 19.0 × 10⁹/L (with neutrophils at 86.4 % and lymphocytes at 8.8 %), platelets at 207 × 10⁹/L, sodium at 138 mmol/L, potassium at 3.09 mmol/L, urea at 6.39 mmol/L, creatinine at 94.69 μmol/L, C-reactive protein at 1.94 mg/L, and Troponin I at 3.4 ng/L. Venous blood gas analysis indicated lactic acidosis with a pH of 7.122, pCO₂ of 75 mmHg, PO₂ of 80 mmHg, bicarbonate levels at 24.8 mmol/L, a base excess of −6.2, and a lactate level of 3.7 mmol/L. A urine toxicology screen returned negative, and blood cultures and nasal swabs for respiratory pathogens were sent for further analysis.

In the emergency department, the patient was diagnosed with acute pulmonary edema due to chlorine gas inhalation. He was initially managed with oxygen therapy at 8 L per minute, but his respiratory distress worsened, necessitating transfer to the ICU within 1 h. Upon ICU admission, the intensivist diagnosed moderate ARDS based on the following criteria: acute onset following chlorine exposure, bilateral lung infiltrates confirmed by chest CT showing consolidation and ground-glass opacities, P/F ratio of 131 mmHg (PaO2: 105 mmHg with FiO2: 80 % via non-rebreather mask), and absence of cardiac origin confirmed by normal echocardiogram findings. The initial working diagnosis was lower respiratory tract infection with wheezing, and treatment was initiated with intravenous ceftriaxone (2 g every 12 hours), ciprofloxacin (2 g every 12 hours), and methylprednisolone (80 mg every 24 hours) was administered for 6 days. Aerosolized bronchodilators were also administered, including salbutamol sulfate and budesonide. Non-invasive ventilation (NIV) in BiPAP mode was applied, with IPAP at 10 mmHg, EPAP at 5 mmHg, and FiO₂ progressively increased from 50 % to 80 %, targeting an SpO₂ of 90 %.

Due to the severity of his condition, particularly the presence of pink, frothy sputum, the decision was made to intubate the patient and initiate mechanical ventilation. Sedation and muscle relaxation were achieved with midazolam0.05 mg/kg/h, fentanyl 1 mcg/kg/h, and rocuronium 0.6 mg/kg bolus followed by 10 mcg/kg/min. He was placed on volume-controlled ventilation (VCV) with a tidal volume of 450 mL, PEEP of 8 cmH₂O, a respiratory rate of 24 breaths per minute, and FiO₂ at 60 %. His pulse rate increased to 150–165 beats per minute, and his blood pressure surged to 180/100 mmHg, prompting the administration of nicardipine at a dose of 5 mg/h to control his hypertension.

The patient's condition remained critical over the first three days, but by the fourth day, he was transitioned from controlled ventilation to CPAP-assisted ventilation via an endotracheal tube. On the fifth day, the patient was extubated and placed on BiPAP ventilation. By the seventh day, he was able to breathe independently and was subsequently discharged from the hospital in a stable condition.

## Data availability

The data in this study are available upon reasonable request.

## Funding

This research received no external funding.

## Declaration of competing interest

The authors declare that they have no known competing financial interests or personal relationships that could have appeared to influence the work reported in this paper.

## Abbreviations

ARDS, Acute respiratory distress syndrome; ICU, Intensive Care Unit; CT, Computed tomography; CRP, C-reactive protein; NIV, non invasive ventilation; BiPAP, bilevel positive airway pressure; PEEP, Positive end-expiratory pressure.
